# Fight Fire with Fire: Detecting Forest Fires with Embedded Machine Learning Models Dealing with Audio and Images on Low Power IoT Devices

**DOI:** 10.3390/s23020783

**Published:** 2023-01-10

**Authors:** Giacomo Peruzzi, Alessandro Pozzebon, Mattia Van Der Meer

**Affiliations:** 1 Department of Information Engineering, University of Padova, 35131 Padova, Italy; 2 Department of Information Engineering and Mathematics, University of Siena, 53100 Siena, Italy

**Keywords:** IoT, AIoT, embedded ML, fire detection, audio signals, image signals, LoRaWAN

## Abstract

Forest fires are the main cause of desertification, and they have a disastrous impact on agricultural and forest ecosystems. Modern fire detection and warning systems rely on several techniques: satellite monitoring, sensor networks, image processing, data fusion, etc. Recently, Artificial Intelligence (AI) algorithms have been applied to fire recognition systems, enhancing their efficiency and reliability. However, these devices usually need constant data transmission along with a proper amount of computing power, entailing high costs and energy consumption. This paper presents the prototype of a Video Surveillance Unit (VSU) for recognising and signalling the presence of forest fires by exploiting two embedded Machine Learning (ML) algorithms running on a low power device. The ML models take audio samples and images as their respective inputs, allowing for timely fire detection. The main result is that while the performances of the two models are comparable when they work independently, their joint usage according to the proposed methodology provides a higher accuracy, precision, recall and F${}_{1}$ score (96.15%, 92.30%, 100.00%, and 96.00%, respectively). Eventually, each event is remotely signalled by making use of the Long Range Wide Area Network (LoRaWAN) protocol to ensure that the personnel in charge are able to operate promptly.

## 1. Introduction

Forest fires are the main cause of desertification, and they have a disastrous impact on agricultural and forest ecosystems. According to the European Forest Fire Information System [1], around 570 km${}^{2}$ of land were destroyed by fire in 2020. Therefore, setting up an automatic detection system for prompt signalling of fire events is of the utmost importance to limit damages and contain the extent of fires. To this end, the present paper shows a prototype of an autonomous Video Surveillance Unit (VSU) system, bearing in mind forests as the deployment scenario, capable of early spotting of fires and consequently signalling alarms by making use of the Long Range Wide Area Network (LoRaWAN) protocol to remotely notify in-charge personnel. The VSU is based on low power Internet of Things (IoT) devices capable of running Machine Learning (ML) algorithms. Indeed, fire recognition is carried out by resorting to two embedded ML models: the former deals with audio signals, while the latter deals with pictures. To this end, the VSU is provided with two on-board microphones and an on-board camera to feed the ML models with data captured by sensors in the deployment environment. The models are trained and tested on specially gathered datasets, along with a test video reproducing the deployment scenarios and the various phenomena that may take place there.

In light of this, the prototype in this work falls within the new paradigm of the Artificial Intelligence of Things (AIoT), which is encountered whenever Artificial Intelligence (AI) meets IoT [2]. In its essence, all of the techniques and models included within AI are inherited and properly adapted to be executed on low computational capability devices aiming at solving the problems [3] such as clustering [4], regression [5], classification [6], and anomaly detection [7].

The motivation of the paper is to propose a VSU prototype enabled by embedded ML algorithms that is capable of timely detection of forest fires and remote signalling of an alarm. Inferences are executed by making use of the data sampled by the on-board sensors (i.e., a camera and two microphones). On the other hand, the main contributions are the following:

- Collect two ad hoc datasets to train and preliminary test the developed ML models. In particular, the former is a dataset containing audio files labelled into two classes, “Fire” and “No-Fire”, on the basis of the presence of fire-related sounds mixed with sundry environmental noises typical of forests (e.g., wind, insects, animals). Conversely, the latter is a dataset containing pictures labelled into the same classes discriminating the presence of fires within forest environments in a wide range of weather conditions and moments of the day (i.e., at night and in daylight).
- Present a ML-enabled embedded VSU prototype that simultaneously runs two classifiers dealing with audio and picture data in turn in order to recognise forest fires.
- Test and select the devised embedded ML algorithms and propose alarm conditions in order to enhance the classification capability of the VSU prototype, and finally measure the latency and current absorption of the prototype.

The technique presented in this paper proposes a novel approach not found in similar works. This aspect is analysed in detail in Section 2; however, to the best of our knowledge no contribution proposing the usage of embedded ML for the detection of fires exploiting both audio and imaging can be found in literature. The rest of the paper is drawn up as follows. Section 2 summarises the current state-of-the art about the topic. In the next Section, the most significant works in the literature dealing with fire detection are presented, and a comparison with the contribution proposed in this work is provided. Section 3 describes the gathered datasets, while Section 4 presents the system architecture of the VSU prototype. Section 5 shows the tests the VSU prototype underwent, while Section 6 highlights conclusions and final remarks, along with suggestions for future works.

## 2. Related Works

Modern fire recognition and warning devices are mainly based on satellite monitoring of risk areas or on the processing of data from sensor nodes arranged on the site of interest, which are sent to central servers for analysis [8]. However, conveying raw data on remote computing architectures implies generating a potentially enormous amount of network traffic, meaning that broadband communication technologies have to be adopted weighing both running costs and hardware power consumption. On the other hand, edge computing paradigms enabling local processing of data to eventually broadcast alarms are far more convenient from such points of view.

Fire detection systems can be set up by making use of an IoT system coping with smoke sensors [9], although such an approach is error prone, as the mere presence of smoke is not sufficient to infer the existence of fires (e.g., extinguished cigarette butts). This drawback can be solved by resorting to data fusion techniques exploiting heterogeneous data, such as the sound of fire coupled with images (as this paper proposes) or by joining smoke sensors with temperature sensors [10]. Data fusion is the paradigm proposed in [11], where fires in indoor environments are spotted by adopting temperature, gas, and light sensors. However, this approach is highly unreliable outdoors (e.g., in forests), leading to its actual impracticability, though a similar contribution can be found in [12] attempting to set up a proof-of-concept for what concerns multi-hop Wireless Sensor Network (WSN) to propagate alarms.

Fire recognition systems with AI features have been proposed as well. For instance, in [13], a VSU was proposed tasked with preventing vandalisms in smart city domains due to the deliberate setting on fire of waste disposal containers. Similarly, [14] proposed a fire recognition system for smart cities which makes use of sensor nodes deployed in the field, unmanned aerial vehicles, and image processing. In the same vein, taking into account forests as application scenario, [15,16] proposed drones as image collectors, on which AI algorithms were applied in order to assess the presence of fires. Although such systems are pervasive, they definitely need more commitment to be installed and run conversely with respect to the VSU prototype proposed herein. Other types of systems based on image processing allow recognition of forest fires thanks to the use of AI algorithms [17]. One of these is the usage of ensemble learning as proposed in [18], exploiting sundry AI models taking as input visual images to make predictions. This is the same approach taken in the present paper through exploiting a Neural Network (NN) dealing both with audio and picture data. Then, concerning data fusion and AI techniques, the simultaneous employment of sundry sensors such as the ones for measuring air quality and particulate, temperature, and humidity was the key point in [19], where all this information was exploited to train an embedded ML model inferring the probability of forest fire. Though this can be a valid alternative, it is dependent on exogenous weather conditions; indeed, in the case where a fire is set far away, the sensor node could output a misclassification if windy currents transport particulate and fire-related gases. On the other hand, the VSU prototype in the present work can counteract this shortcoming, as it takes as input only audio and pictures, which are intrinsically associated with the deployment site. In the same way, [20] solves this weak point through an AI algorithm dealing with images which is grounded on colour probability models based on the acquired pictures; however, no audio is sampled. Moreover, although the method proposed in [20] can be potentially scaled to be run on embedded platforms, it is energy-hungry with respect to the technique in the present paper, as it continuously relies on visual data that needs to captured by cameras. On the other hand, fire recognition systems only exploiting audio signals can be found in [21], where classification was performed on the cloud network, which would add overhead and latency with respect to carrying out classification at the edges of the network, as the VSU prototype of this paper performs. In addition, as described in Section 5, the joint use of NN models in dealing with audio and pictures is more effective in recognising fires than the exploitation of visual and audio classifiers on their own, as confirmed by comparing the results of [20,21] with the results of this paper. Likewise, related works facing the problem with ML models taking as input only images are countless [22,23,24,25]. Conversely, the literature on forest fire detection systems leveraging embedded ML algorithms grounded on audio data is more scarce. Indeed, the only related work available is [26], which follows a similar methodology with respect to that in this paper; however, owing to the fact that no images are analysed, several misclassifications are provided. In particular, the misclassification rate is comparable with that of the ML model dealing with audio signals of this paper, and is higher than that of the VSU prototype additionally adopting the ML model analysing images.

Embedded ML has gained momentum, becoming a hot topic. Indeed, sensor nodes enabled with embedded ML are able to perform local data processing on the measurements from on-board transducers, allowing them to extract useful information without the need to transmit raw data to remote data centres for analysis. Related works proposing embedded ML models dealing with audio and images can be found for a wide set of application scenarios. For instance, in [27], a VSU for intrusion detection was proposed; similarly, [28] presented a face recognition system. On the other hand, embedded ML models can be fundamental for visually impaired people [29] in order to provide guiding support, and even in the military field in order to spot sea mines [30]. Conversely, embedded ML systems coping with audio data have been widely adopted in the healthcare domain. For example, in [31] the authors conceived a methodology for early diagnosis of sleep bruxism without the necessity for patients to be monitored within specialised sleep laboratories. In the same way, [32,33] put forth tools for remote detection of COVID-19 infections by classifying coughing and breathing. Audio-based embedded ML models have found application in different domains as well, including urban sound identification [34], detecting mosquito populations [35], and anomaly detection purposes [36].

In light of the above literature review, and to the best of the authors’ knowledge, a system such as the one proposed in this paper is not currently available. Indeed, although there are plenty of devices for detecting forest fires from acquired images, and there is at least one proposal for the same task exploiting audio samples, there is currently a gap in IoT devices with embedded ML capabilities for identifying forest fires by simultaneously making use of images and acoustic signals gathered by on-board sensors.

## 3. Datasets Description

With the aim of recognising forest fires, two datasets were created: one containing audio files, and one containing images. Of course, both of the datasets included audio and pictures related to forest contexts (e.g., animal sounds, wind, fire, woods, etc.).

### 3.1. Audio Dataset

Fire sound recognition in forest environments can be traced back to a classification problem, in which the classifier receives audio signals as inputs and returns the probability that the sample at hand belongs to each of the classes. To this end, two classes were defined: “Fire” and “No-Fire”. For each of them, audio samples were collected in an unbiased and heterogeneous way in order to train the network to distinguish as many inputs as possible. In other words, audio contained the sound of fire mixed with noises related to climatic events, to fauna, or even to unexpected events such as the passage of aeroplanes, as well as similar noises devoid of the sound of fire. Concerning the source of the samples, they were retrieved from the openly available FSD50-K [37] and ESC-50 [38] datasets, while others were specially recorded by making use of an Audio-Technica AT2020 microphone driven by a Behringer UM2 sound card and a PC running Audacity. Such samples were recorded at 44 kHz sampling frequency, while the microphone was placed from 2 m to 10 m distance from the fire. Moreover, the duration of each audio sample included in the dataset was standardised to 5 s to improve the training of the relative NN, and in view of the VSU embedded microphone each file was resampled at 16 kHz.

The audio dataset was composed of 2864 samples, divided as shown in Table 1, and can be retrieved at [39].

Audio samples belonging to the class “Fire” can be characterised as follows according to their audio type:

- Clean Fire—the sound of fire was the only audible noise
- Fire with Animal Sounds—the sound of fire was superimposed on sounds coming from animals living in forests (e.g., birds)
- Fire with Insect Sounds—the sound of fire was superimposed on sounds coming from insects living in forests (e.g., cicadas)
- Fire with Wind Noise—the sound of fire was superimposed on the noise of wind or of shaking leaves
- Additional Fire Samples—digitally mounted samples in which the sound of fire was superimposed on the samples belonging to the “No-Fire” class with different intensity levels, with the aim of improving generalisation capability of the relative trained NN
- Recordings—samples recorded according to the procedure explained above during the combustion of plant residues at a variable distance from the flames.

Audio samples belonging to the class “No-Fire” can be characterised as follows according to their audio type:

- Animal Sounds—samples of sounds from animals living in forests (e.g., birds) that were not exploited as background for the “Fire with Animal Sounds” typology of the “Fire” class
- Insect Sounds—samples of sounds from insects living in forests (e.g., cicadas) that were not exploited as background for the “Fire with Insect Sounds” typology of the “Fire” class
- Wind Noise—samples of wind noise or of shaking leaves that were not exploited as background for the “Fire with Wind Noise” typology of the “Fire” class
- Rain Noise—samples of rain noise or thunder
- Sundry Samples—a set of samples belonging to events that are marginal with respect to the context of interest, but which could be recorded by the device (e.g., sounds produced by agricultural machinery, the sound of aircraft, the buzz of people talking in the distance), included in order to augment the generalisation capability of the relative trained NN.

### 3.2. Picture Dataset

The picture dataset was collected in the same fashion as the audio dataset. Specifically, all the possible scenarios in which the prototype VSU might work were considered; thus, pictures of forests in sundry weather conditions (e.g., clear, rain, fog, etc.) as well as in different seasons and time of the day were gathered to account for several light conditions. Pictures belonging to the “Fire” class show burning forests both at night and in daylight. Concerning the source of the pictures forming the dataset, some were retrieved from the openly available dataset [40], while others were specially taken by exploiting the camera embedded in the prototype VSU. Moreover, bearing in mind this camera, the pictures were resized at 320×240 px and converted to greyscale.

The picture dataset was composed of 5060 samples, divided as shown in Table 2, and can be retrieved at [41].

Pictures of the “Noise” type include greyscale samples as well as samples captured by covering the camera of the prototype VSU with various objects. These were added to train the relative NN to distinguish any obstacle that might interfere with the camera field of view.

## 4. System Overview

**Figure 1:**
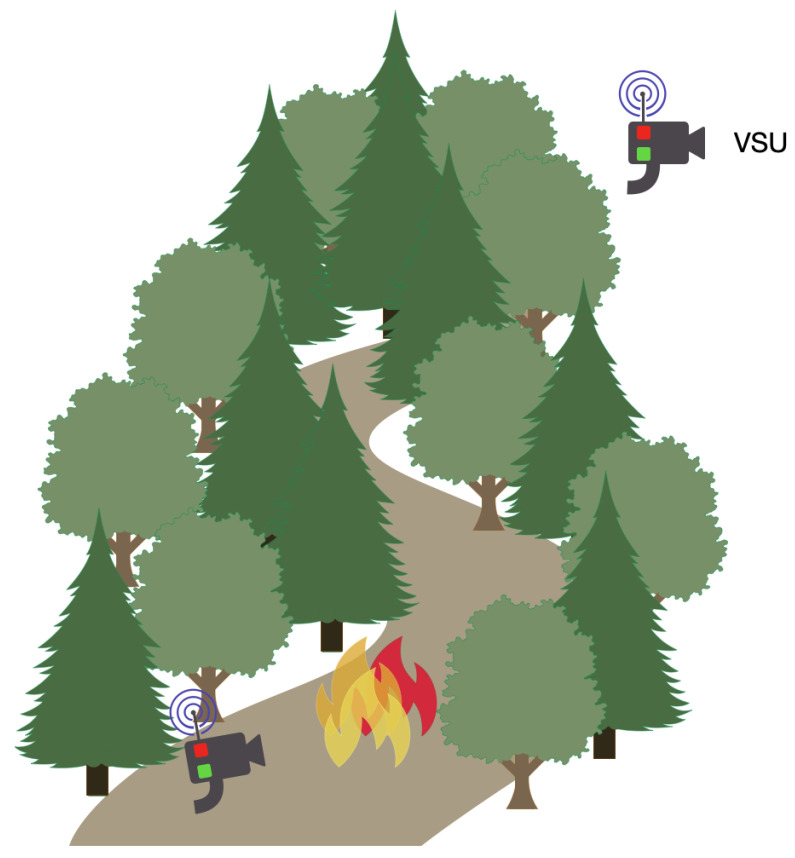
Application scenario of the prototype VSU.

The application scenario for which the prototype of VSU was designed is depicted in Figure 1, which is a forest context that may be potentially at risk of fire. To this end, the prototype VSU aims at preventing and limiting the extent of possible danger by early detection of any fire occurrence and prompt signalling via LoRaWAN links. For this purpose, the VSU possesses embedded ML algorithms capable of spotting fires by making use of both audios and pictures directly recorded by on-board sensors. All of these were developed in TensorFlow by means of the Keras Python library. In addition, the VSU is provided with a LoRaWAN transceiver to send alarms whenever the start of a fire is recognised. The VSU continuously runs a NN devoted to spotting fires by relying on the captured audio samples; in case of a positive fire inference, it resorts to another NN whose objective is to detect fires by exploiting pictures taken with the on-board camera. At last, a fire alarm is sent via LoRaWAN. Finally, in order to preserve privacy and avoid related issues, neither audio nor pictures are stored or broadcast, as they are locally recorded and analysed, then eventually deleted. The hardware composing the VSU prototype is presented in Section 4.1, the NNs are described in Section 4.2 and Section 4.3, respectively, and the overall functioning of the scheme is shown in Section 4.4.

### 4.1. Hardware

**Figure 2:**
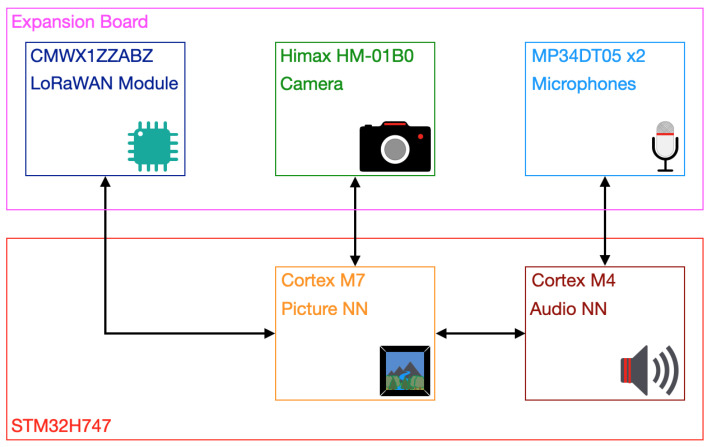
VSU prototype block diagram.

Figure 2 shows the block diagram of the VSU prototype. Its core is an STM32H47 microcontroller, produced by STMicroelectronics, which is a dual-core microcontroller with enough computational power to run embedded ML algorithms. Moreover, the microcontroller was embedded in a development board on which an expansion board containing peripherals was stacked. It includes two microphones (the MP34DT05 produced by STMicroelectronics), a camera (the HM-01B0 produced by Himax), and a LoRaWAN module (the CMWX1ZZABZ produced by Murata) driven by the microcontroller. Its specific cores are a Cortex M4 and Cortex M7, each of which has its own tasks to carry out. The Cortex M4 is devoted to running the audio NN and driving the microphones to feed inputs to the relative NN. On the other hand, the Cortex M7 runs the picture NN and controls the camera that takes the pictures used as input to the related NN, and additionally drives the LoRaWAN module to eventually transmit fire alarms. This choice was motivated by the fact that the audio NN is far tinier and easier to run than the picture one. Therefore, the latter requires a more powerful core to be executed (i.e., the Cortex M7). As concerns the LoRaWAN packets, their payloads contained the mean of the probability of fire, namely p(f), which is related to the inferences generating the fire alarm, to give personnel more insight. Moreover, regarding the LoRaWAN network, for an in-depth description readers may refer to a previous work [42], as the same network prototype is exploited. It is composed of LoRaWAN concentrators receiving LoRaWAN packets sent by the the VSU prototype. These data are demodulated by the concentrators and then forwarded to a remote network server and an application server running on the cloud by making use of the Message Queue Telemetry Transport (MQTT) protocol.

### 4.2. Embedded ML Model: Audio NN

Two audio ML models, called audio NN #1 and audio NN #2, were designed and developed to compare their performances and accordingly select one of them to be deployed on the Cortex M4 core of the microcontroller. The comparison leading to the choice is described in Section 5.3. Both of them accounted for a preprocessing stage with the objective of extracting features from data and an NN classifier distinguishing whether or not the audio sample at hand belongs to the class “Fire”. The deployed model needs to predictions based on the audio recorded by the on-board microphones. Similarly, both were designed, trained, and subjected to preliminary testing on the audio dataset described in Section 3.1. Furthermore, because data-driven techniques were exploited, a trial-and-error procedure was followed throughout the design of the models. The hyperparameters of both audio NN models and their training parameters are listed in Table 3.

#### 4.2.1. Audio Feature Extraction

Digital microphones output time series data, requiring the adoption of sliding temporal windows during processing. Specifically, audio NN #1 had a window size of 4 s and a sliding step of 2 s, while audio NN #2 had a window size of 2 s and a sliding step of 2 s. This choice entailed multiple overlapping windows, resulting in a data augmentation scheme leading to the expansion of the audio dataset to 5720 and 8580 samples for audio NN #1 and audio NN #2, respectively.

Feature extraction was carried out by exploiting Mel-scaled spectrograms for each of the temporal windows. This choice was motivated by the proven effectiveness of this technique when exploited for non-voice audio data, for instance, in the healthcare domain [31,43,44,45], for sound detection [46], and in robotic interfaces [47]. Mel-scaled spectrograms are spectrograms undergoing Mel filterbanks, in which a series of triangular filters reduce the correlation between consecutive frequency bins of the spectrogram to which they are applied. In other words, Mel-scaled spectrograms are derived from linear spectrograms by passage through Mel filterbanks according to the Mel scale. This translates into the fact that each triangular filter has a maximum unitary response at its central frequency that linearly decreases towards a null response in correspondence with the central frequencies of the two adjacent filters. Specifically, the Mel scale was first exploited to measure the perception of the pitch of sounds according to listeners’ judging them to be equal in distance from one another. In particular, a 1000-mel pitch corresponds to a 1 kHz tone at 40 dB above the listener threshold, meaning that a given frequency *f* translates to a mel *m* as

(1) $$m=2595\;log_{10}\left(1+\frac{f}{700}\right).$$

From a practical perspective, Mel filterbanks are able to extract more features from low frequencies than from higher ones, with the aim of replicating the nonlinear behaviour of the human ear.

Spectrograms were derived from temporal frames on which the Fast Fourier Transform (FFT) was applied. Specifically, both audio NN #1 and audio NN #2 had temporal frames of 0.05 s, overlapping 0.025 s with the next to achieve a 50% superimposition. Then, because audio was resampled at 16 kHz, in order to match with the sampling frequency of the on board microphones of the VSU prototype we ensured that each temporal frame contained 800 samples, translating to a 1024-point FFT. In addition, both audio NN #1 and audio NN #2 adopted 40 filterbanks applied to the spectrograms, with 300 Hz as the lowest frequency band edge of the first filterbanks. Eventually, audio NN #1 had a threshold of −52 dB as the noise floor, while the respective value was −72 dB for audio NN #2.

#### 4.2.2. Convolutional Neural Network Classifier

Audio NN #1 has a mono-dimensional Convolutional Neural Network (CNN) as classifier, with two outputs representing the probability that a given input belongs to the “Fire” or “No-Fire” class. This type of NN was chosen because of its ability to deal with features arranged in spatial domains as images, including spectrograms. The classifier had a reshape layer at its input that sorts the extracted features from data and forwards them to the next layers. Then, a mono-dimensional layer containing 8 neurons was inserted, followed by a pooling layer, to limit the model dimension by computing the maximum value stemming from the previous convolutional layer. This means that, bearing in mind the model deployment on the Cortex M4 core of the microcontroller, a nonlinear down-sampling was carried out trading off the required computational power and model complexity for accuracy. Next, a second block mono-dimensional convolutional layer having 16 neurons was placed, followed by a pooling layer identical to the previous one. In addition, all the neurons in the convolutional layers used the Rectified Linear Unit (ReLU) as the activation function. The last layers of the classifier were a flattening layer to properly rearrange data as input to a dropout layer having rate of 0.5, which was exploited so to reduce the risk of overfitting during training owing to the fact that a random fraction of the network connections was pruned throughout the training, followed by a fully connected layer of 64 ReLU neurons, followed by another dropout layer with a rate of 0.5 and a softmax layer to provide the output class probabilities as the result.

Audio NN #2 is more complex; it has a two-dimensional CNN with the same number of outputs as the first model. It then includes a reshape layer followed by four blocks of two-dimensional convolutional and pooling layers. The latter compute the maximum value in the same way as in the other model. On the other hand, the convolutional layers contain 8, 16, 32, and 64 ReLU neurons in turn. The final stage of the classifier is the same as audio NN #1, that is, a dropout layer with a rate of 0.5, a fully connected layer of 64 ReLU neurons, another dropout layer with a rate of 0.5, and a softmax layer.

Both models were trained by adopting the standard training–validation procedure exploiting the standard back-propagation algorithm, employing a learning rate of 0.005. In particular, the Categorical Cross-Entropy (CCE) was adopted as the loss function:

(2) $$CCE=-{\underline{y}}^{T}\cdot log\left(\underline{\hat{y}}\right)$$

where $\underline{y}$ is the target label input and $\hat{y}$ is the related label for the network input at hand. For the sake of the training process, the audio dataset was divided into training, validation, and test subsets according to the respective rates of 0.6, 0.2, and 0.2. Accordingly, the examples belonging to both classes (i.e., “Fire” and “No-Fire”) were split in the same fashion.

#### 4.2.3. Model Quantisation for Deployment

The general ML model needed to be converted into a more compact version, resulting in an embedded ML model able to be run on a microcontroller. This procedure was carried out by resort to the TensorFlow Lite framework, transforming a ML model into a C++ library able to be included in the microcontroller firmware and compiled for deployment. In particular, both the audio NN models were converted into quantised versions accounting for only 8 bit integers in order to optimise memory occupancy in the microcontroller. Hereinafter, only the 8 bit versions are considered in the tests and performance comparison used to select one of the two models.

### 4.3. Embedded ML Model: Picture NN

The picture NN model was grounded on the MobileNetV2 classifier [48], which is an open source CNN specially developed by Google to run on embedded devices. Thus, it is suitable for deployment on the Cortex M7 core of the microcontroller. The picture NN model has a feature extraction stage and a classification stage aiming at distinguishing the input pictures on the basis of whether they contain fires in forests, meaning that the sample at hand belongs to the “Fire” class or does not, in which case the sample belongs to the “No-Fire” class. The model was designed, trained, and preliminarily tested on the greyscale version of the picture dataset described in Section 3.2, as the camera on board the VSU prototype cannot take colour images. In addition, because data driven techniques were employed, a trial-and-error procedure was adopted during the design of the model. As is explained later, the picture NN was in charge of validating the output provided by the audio NN.

#### 4.3.1. Picture Feature Extraction

Due to the memory limits imposed by the hardware on which the model was deployed, and in view of the fact that the Cortex M7 core of the microcontroller has to deal with LoRaWAN connectivity, the picture NN took as input resized samples from 320×240 px (i.e., the on-board camera resolution) to 96×96 px. Of course, as these proportions could not be maintained, the pictures underwent a squash process.

Owing to the characteristics of the on-board camera, the input samples were in greyscale. This facilitated the feature extraction process, as the features could be represented by the grey level of each pixel of the sample.

#### 4.3.2. Convolutional Neural Network Classifier

As previously stated, MobileNetV2 was adopted as the classifier for the picture NN. MobileNet is a depthwise CNN that can significantly reduce the number of parameters needed to process images compared to a typical CNN used for the same purpose, and is specially devised to be run on embedded devices. Because it is a pre-trained model making use of extracted features from large datasets of images belonging to numerous recognition problems, a transfer learning step is sufficient, thereby saving time compared to training the whole architecture. In contrast with the adopted methodology for the design phase of the audio NN, different architectures were not explored for the picture NN, as MobileNetV2 largely proved to be effective for the classification problems to be carried out by low computational power devices, as can be verified from the literature. Indeed, the application scenarios are countless, including ensuring security in accesses to public places [49], image processing [50], cactaceae detection [51], analysing the quality of corn [52], waste management [53,54], body temperature and face mask detection [55], and food classification [56].

The model was trained by resort to the training–validation procedure by making use of the back-propagation algorithm, adopting a learning rate of 0.000045. Moreover, the CCE was used as the loss function (see Equation (2)). In addition, in order to perform training the greyscale version of the picture dataset was split into training, validation, and test subsets according to the rates of 0.6, 0.2, and 0.2, respectively. Consequently, the examples belonging to the two classes of “Fire” and “No-Fire” were divided in the same fashion.

#### 4.3.3. Model Quantisation for Deployment

As stated in Section 4.2.3, the conversion of a ML model meant to be deployed and run on an embedded device is a crucial step. The procedure explained above was adopted for the picture NN, that is, it was converted into a C++ library by making use of TensorFlow Lite. Then, the library was included in the microcontroller firmware to be compiled and deployed on the device. The picture NN was converted into its version accounting for only 8 bit integers, as optimising memory occupancy was of the utmost importance because the Cortex M7 core of the microcontroller has to additionally deal with LoRaWAN transmissions.

### 4.4. VSU Functioning Scheme

**Figure 3:**
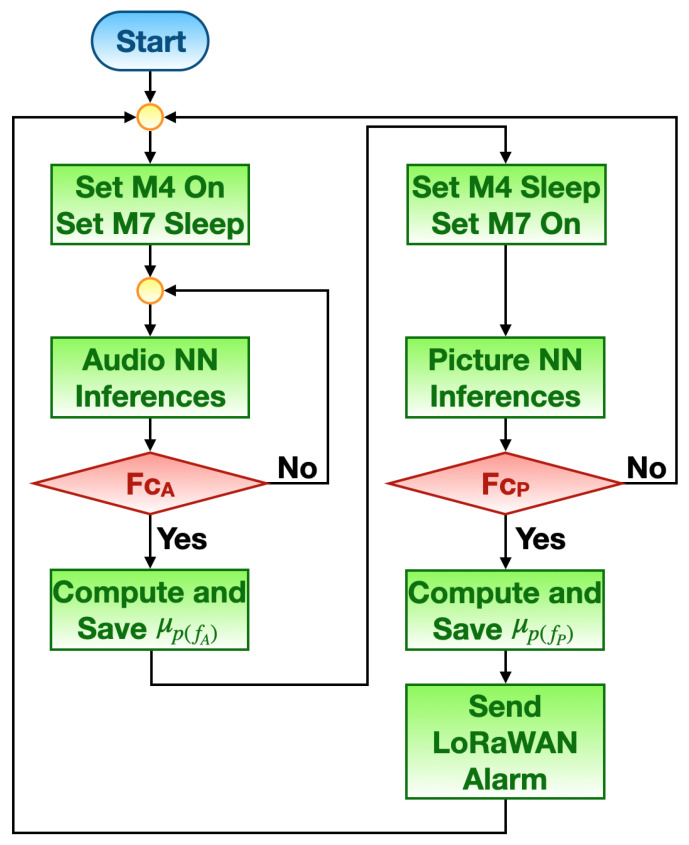
VSU prototype functioning flow chart.

The VSU prototype worked by following the functioning scheme shown in Figure 3. For the time being, without loss of generality, suppose that one of the two audio NN models has been chosen; the detailed procedure is explained in Section 5.3.

First, the Cortex M4 core of the microcontroller is turned on, while the Cortex M7 core is put into sleep mode to reduce power consumption. Then, the audio NN is run to provide inferences on the signals recorded by the on-board microphones. Let $Fc_{A}$ be the condition under which the outputs provided by the audio NN can be associated with a fire alarm ($Fc_{A}$ is described in Section 5.3). If $Fc_{A}$ is not met, then the audio NN continues to infer; otherwise, the mean value of the probability of fire related to the outputs of the audio NN generating $Fc_{A}$, namely, $\mu_{p(f_{A})}$, is computed and stored. At this stage, the result provided by the audio NN is validated by making use of the picture NN; therefore, the Cortex M4 core of the microcontroller is put into sleep mode to reduce power consumption, while the Cortex M7 core is turned on in order to run the picture NN that classifies the pictures taken by the on-board camera. Let $Fc_{P}$ be the condition under which the outputs provided by the picture NN can be associated with a fire alarm ($Fc_{P}$ is described in Section 5.3). If $Fc_{P}$ is not met, the VSU prototype restarts its workflow by turning the Cortex M4 core on and putting the Cortex M7 into sleep mode; otherwise, the mean value of the probability of fire related to the outputs of the picture NN generating $Fc_{P}$, namely, $\mu_{p(f_{P})}$, is computed and stored. This condition translates into the actual risk of fire, denoting the need for signalling said phenomenon. To this end, $\mu_{p(f_{A})}$ is recalled and paired with $\mu_{p(f_{P})}$ in order to form the payload of a LoRaWAN packet to be remotely sent. Finally, the VSU prototype restarts its workflow by turning on the Cortex M4 core and putting the Cortex M7 core into sleep mode.

## 5. Laboratory Tests and Results

The laboratory tests conducted on the VSU prototype aimed at the following objectives:

- Assessing the classification performance on the relative test set for audio NN #1, audio NN #2, and the picture NN;
- Assessing the hardware performance of audio NN #1, audio NN #2, and the picture NN;
- Selecting one model between audio NN #1 and audio NN #2 on the basis of their performance on an ad hoc edited video;
- Choosing $Fc_{A}$ and $Fc_{P}$ on the basis of the performance of the selected audio NN and the picture NN on the same video mentioned above;
- Assessing the performance of the VSU prototype from the point of view of classification and latency, making use of the same video as above;
- Measuring the current drawn by the VSU prototype.

### 5.1. Performance on Test Set and Hardware

**Figure 4:**
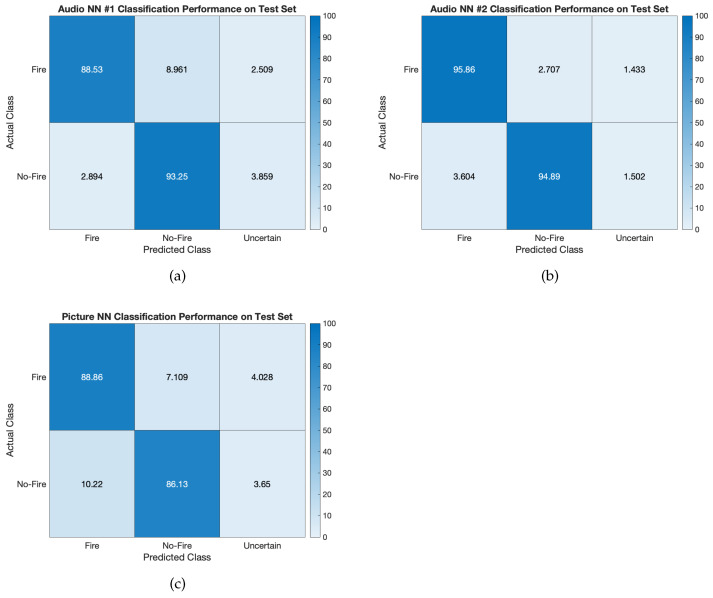
Confusion matrices showing the performance of the models on the test set: (**a**) audio NN #1, (**b**) audio NN #2, and (**c**) picture NN.

A preliminary test consisted of assessing the classification accuracy on the test set. To this end, audio NN #1, audio NN #2, and the picture NN were provided with samples belonging to the relative test sets as inputs, derived from the 0.2 ratio of the audio dataset (see Section 3.1) for audio NN #1 and audio NN #2 and from the picture dataset (see Section 3.2) for the picture NN. The results are reported in Figure 4 in the form of confusion matrices.

Audio NN #1 had an overall accuracy of 90.890%, audio NN #2 had 95.375%, and the picture NN had 87.495%, meaning that all the model showed a satisfactory generalisation capability stemming from an adequate training stage. As concerns True Positives (TPs) and True Negatives (TNs) classification, audio NN #1 achieved 88.53% and 93.25%, audio NN #2 95.86% and 94.89%, and the picture NN 88.86% and 86.13% respectively. Conversely, regarding misclassifications, the False Positive (FP) and False Negatives (FN) rates were 2.894% and 8.961% for audio NN #1, 3.604% and 2.707% for audio NN #2, and 10.22% and 7.109% for the picture NN, respectively. On the whole, misclassifications were limited; that said, the most dangerous outcomes are related to FNs, as this means that an actual fire occurred and the model did not recognise it. These instances can potentially be reduced by a model accounting for audio and pictures simultaneously, as the proposed VSU prototype is intended to operate. Indeed, the next tests aimed to assess the behaviour of the VSU prototype according to the procedure described in Section 4.4. In addition, when the output class probability is between 0.4 and 0.6 an uncertain outcome is provided, meaning that the model result is not reliable. This phenomenon does not take place often, occurring no more than in 4.028% of cases for the picture NN, 3.859% for audio NN #1, and 1.502% for audio NN #2.

From the hardware perspective, Table 4 summarises the three models. Owing to the deployment of the models on the microcontroller, it is of the utmost importance to minimise RAM and flash occupancy in order to allow their execution. Specifically, the microcontroller features 2 MB of flash memory and 1 MB of RAM, and both these memories are shared among the two cores. However, figures in Table 4 suggest that the ML models can be correctly executed along with the sundry routines (e.g., initialization, data transmission, etc.) that the microcontroller has to carry out. Moreover, in order to limit the current draw of the VSU prototype, the execution time is important, as it is directly proportional to current drawn. Therefore, focusing solely on hardware specifics, audio NN #1 is preferable to audio NN #2 despite the latter being slightly more accurate.

### 5.2. Ad Hoc Edited Test Video

Due to the obvious inability of setting fires in either the laboratory or within real forests, an ad hoc video was specially edited for testing purposes; it is available at [57]. It is an hour-long slideshow consisting of alternating pictures taken at different times of the day every 30 s, to which sounds were added. Pictures and sounds were matched to the displayed scenario, meaning that if no fire was present, audio samples not related to fire were reproduced, while if a fire was present the audio samples related to fire were inserted. The audio and pictures used to test the generalisation capabilities of the VSU prototype did not belong to the datasets described in Section 3. The video was sorted into 26 sections, including equally distributed scenarios belonging to both classes (i.e., “Fire” and “No-Fire”). Moreover, the 13 parts belonging to the “Fire” class were further categorised into 3 typologies according to the extent of fire within the picture and in the corresponding reproduced sound. The first test was a Low Noise Test (LNT), in which audio samples belonging to the “Fire” class were mixed with audio samples belonging to the “No-Fire” class by setting the volume of the “Fire” samples to double that of the “No-Fire” samples. Regarding the images, they had fire in the foreground. The second test was the Mild Noise Test (MNT), in which audio samples belonging to the “Fire” class were mixed with audio samples belonging to the “No-Fire” class by setting both volumes at the same level, while the pictures had clearly visible fires of modest size. The third test was the Extreme Noise Test (ENT), in which audio samples belonging to the “Fire” class were mixed with audio samples belonging to the “No-Fire” class by setting the volume of the “Fire” samples to the half that of the “No-Fire” samples. Concerning the images, they had fires either of a smaller size or far in the background.

### 5.3. Performance on Test Video, Audio NN Model Selection, and Condition of Fire Definition

The objectives of these tests were multiple. For the audio NN models, the tests were used to select one model between audio NN #1 and audio NN #2 and to define $Fc_{A}$. Conversely, for the picture NN only the definition of $Fc_{P}$ needed to be accomplished.

The models were deployed on the microcontroller, and the VSU prototype was tested on the test video described in Section 5.2 by assessing the performance of each of the models separately. The tests were carried out in a dark environment where the only light source was a 24-inch full HD monitor. Audio was reproduced by exploiting a system of four 40 W loudspeakers arranged at the corners of a square having sides 2 m in length. In particular, one of the sides hosted the monitor and the VSU was placed in the centre of the square. Then, the video was reproduced three times (i.e., once for each of the embedded ML models) and the predictions of each model (i.e., the probability that the acquired sample belonged to the “Fire” class) were monitored and stored using a PC acting as a data-logger.

The methods used to define $Fc_{A}$ and $Fc_{P}$ are analogous; therefore, without loss of generality, we only focus on $Fc_{A}$ here. As it is advisable to take into account more than a single inference in order to enhance the reliability of predictions, let $n_{A}$ be the number of consecutive inferences such that $p(f_{A})$ (i.e., the probability that the input audio sample at hand belongs to the “Fire” class) is greater or equal to $th_{A}$ (i.e., the minimum $p(f_{A})$ for which the output of the audio NN model can be interpreted as belonging to the input audio sample in the “Fire” class). Then, let $min_{TP}\left(th_{A}\right)$ be the minimum number of consecutive inferences related as TPs for a given $th_{A}$, and let $MAX_{FP}\left(th_{A}\right)$ be the maximum number of consecutive inferences related as FPs for a given $th_{A}$. The objective is to identify the maximum ${th_{A}}^{*}$ resulting

(3) $$min_{TP}\left({th_{A}}^{*}\right)>MAX_{FP}\left({th_{A}}^{*}\right)+1,$$

where the right-hand side is increased to follow a conservative approach. This means that

(4) $${th_{A}}^{*}=\left\{MAX\left(th_{A}\right):min_{TP}\left(th_{A}\right)>MAX_{FP}\left(th_{A}\right)+1\right\}.$$

Then, ${n_{A}}^{*}$ can be retrieved by considering that if

(5) $${n_{A}}^{*}\geq MAX_{FP}\left({th_{A}}^{*}\right),$$

no FPs are generated, while FNs (i.e., the most dangerous outcomes) are avoided if

(6) $${n_{A}}^{*}\leq min_{TP}\left({th_{A}}^{*}\right),$$

meaning that conditions (5) and (6) merge into

(7) $${n_{A}}^{*}=\left\{n_{A}:MAX_{FP}\left({th_{A}}^{*}\right)\leq n_{A}\leq min_{TP}\left({th_{A}}^{*}\right)\right\}.$$

In light of this, $Fc_{A}$ is the pair ${th_{A}}^{*}$ and ${n_{A}}^{*}$, while $Fc_{P}$ is the pair ${th_{P}}^{*}$ and ${n_{P}}^{*}$.

**Figure 5:**
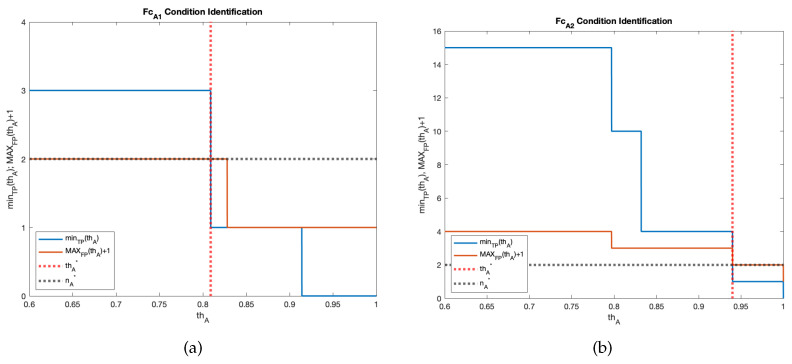
$Fc_{A}$ condition identification: (**a**) audio NN #1 and (**b**) audio NN #2.

Concerning the audio NN models, $Fc_{A}$ for the audio NN #1 (i.e., $Fc_{A_{1}}$), and $Fc_{A}$ for the audio NN #2 (i.e., $Fc_{A_{2}}$) were found by checking conditions (4) and (7) by exploiting the test video described in Section 5.2 and having resort to the the aforementioned test setup. In particular, the relative $min_{TP}\left(th_{A}\right)$ and $MAX_{FP}\left(th_{A}\right)$ for each model were evaluated for $th_{A}$ by varying 0.6 to 1 with steps of 0.001; it would make no sense to analyse the model behaviours for $th_{A}<0.6$, as the classifier would not output a “Fire” result in any case. Moreover, the relative ${th_{A}}^{*}$ and ${n_{A}}^{*}$ forming $Fc_{A_{1}}$ and $Fc_{A_{2}}$ where graphically identified, as shown in Figure 5, where the terms of Equation (3) are plotted. This test proved that for $Fc_{A_{1}}$, ${th_{A}}^{*}=0.808$ and ${n_{A}}^{*}=2$, while ${th_{A}}^{*}=0.940$ and ${n_{A}}^{*}=2$ for $Fc_{A_{2}}$.

**Figure 6:**
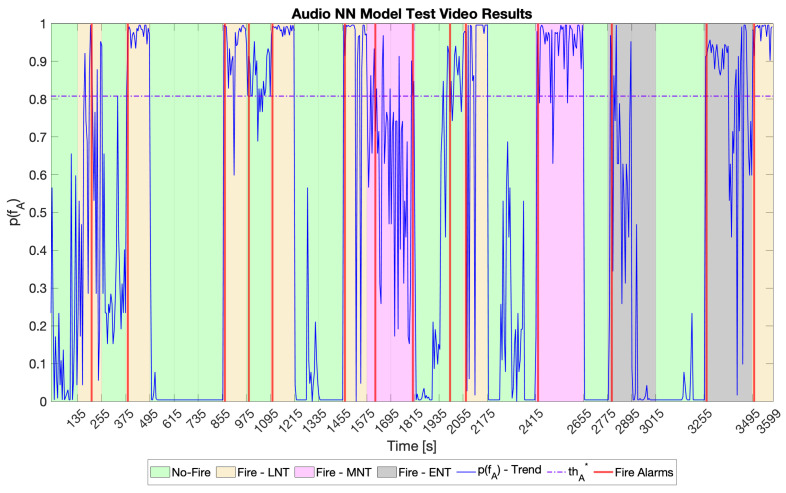
Audio NN model results on the test video. The green areas represent input samples belonging to the “No-Fire” class. The yellow, pink, and grey areas represent input samples belonging to the “Fire” class in LNT, MNT, and ENT, respectively. The solid blue line stands for the trend of $p(f_{A})$, while the dash-dotted line represents ${th_{A}}^{*}$. Finally, the vertical red lines mark the instants at which the audio NN model signalled a fire alarm.

Then, model selection was carried out by looking at the accuracy, recall, precision and F${}_{1}$ score metrics on the test video for the two models considering $Fc_{A_{1}}$ and $Fc_{A_{2}}$, respectively. The results are shown in Table 5, showing that the two models performed the same. Therefore, because the microcontroller has a limited amount of memory and its Cortex M4 core has to deal with other routines, audio NN #1 was preferred thanks to its being better from the hardware perspective (see Table 4). Hereinafter, this model is referred as the audio NN model; its output in terms of $p(f_{A})$ for the test video is displayed in Figure 6.

**Figure 7:**
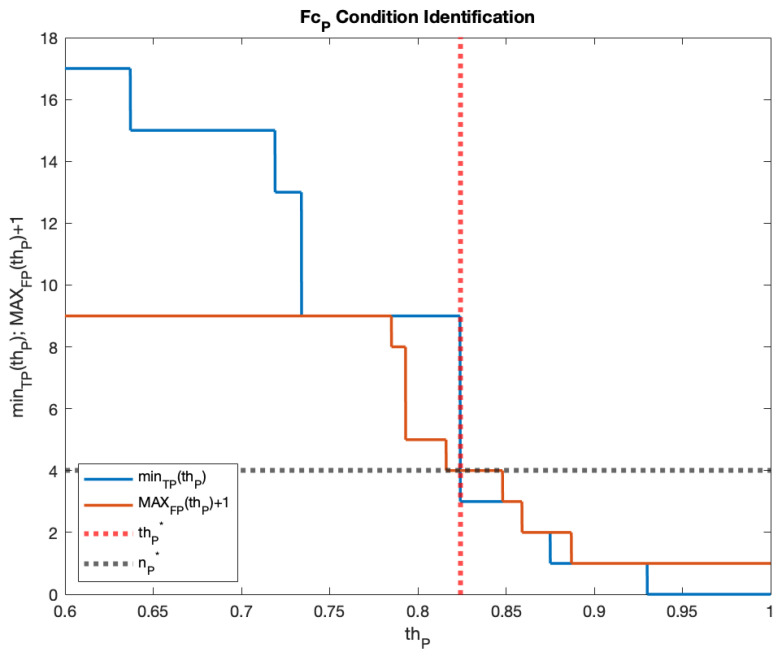
$Fc_{P}$ condition identification.

**Figure 8:**
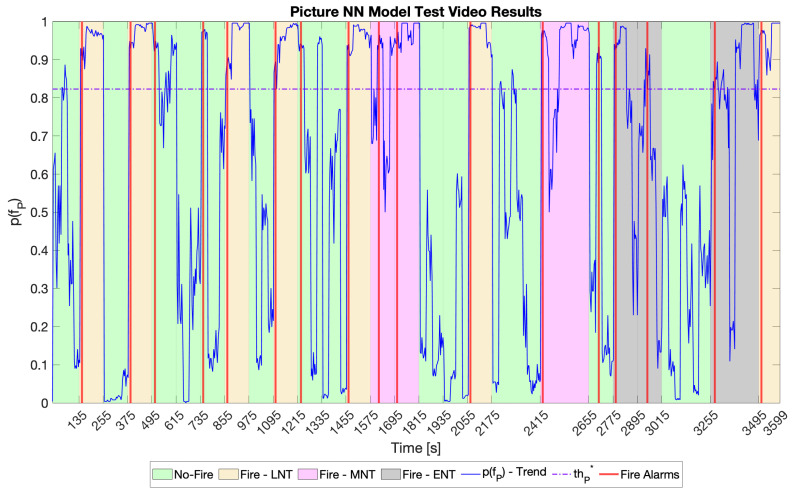
Picture NN model results on the test video. The green areas represent input samples belonging to the class“No-Fire”. The yellow, pink, and grey areas represent input samples belonging to the “Fire” class in LNT, MNT, and ENT, respectively. The solid blue line stands for the trend of $p(f_{P})$, while the dash-dotted line represents ${th_{P}}^{*}$. Finally, the vertical red lines mark the instants at which the picture NN model signalled a fire alarm.

Regarding the picture NN, $Fc_{P}$ was found by adopting the same methodology as that used for the audio models. Figure 7 reports the results, showing that $Fc_{P}$ had ${th_{P}}^{*}=0.823$ and ${n_{P}}^{*}=4$. Table 6 and Figure 8 show the picture NN model results on the test video.

### 5.4. Performance of the VSU Prototype

**Figure 9:**
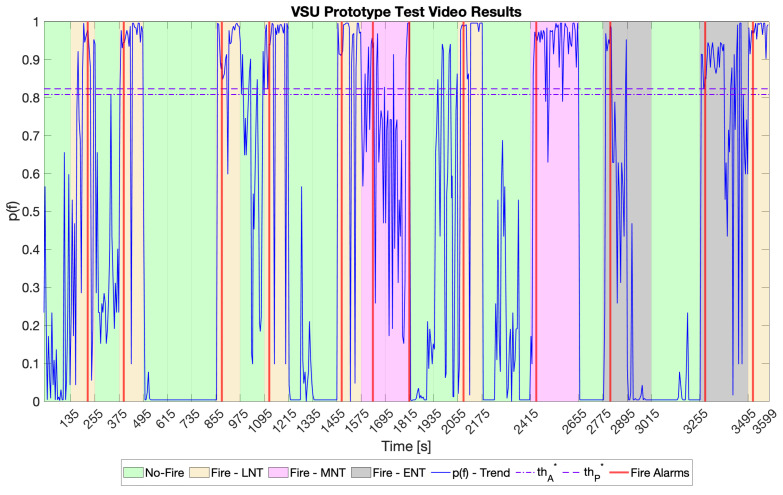
VSU prototype results on the test video. The green areas represent input samples belonging to the “No-Fire” class. The yellow, pink, and grey areas represent input samples belonging to the “Fire” class in LNT, MNT, and ENT, respectively. The solid blue line stands for the trend of p(f), the dash-dotted line represents ${th_{A}}^{*}$, and the dashed line represents ${th_{P}}^{*}$. Finally, the vertical red lines mark the instants at which the VSU prototype signalled a fire alarm.

**Figure 10:**
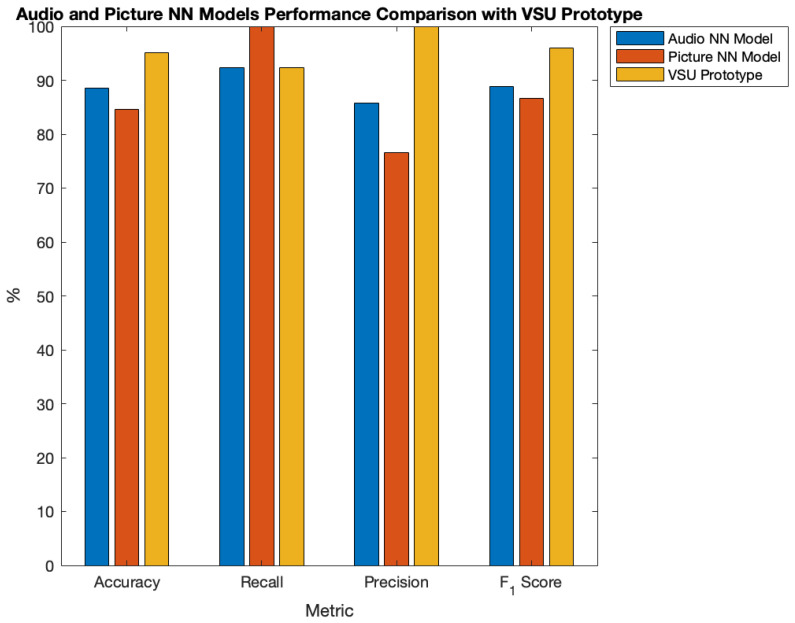
Performance comparison of the audio NN model, picture NN model, and VSU prototype.

After identifying the best audio NN model, $Fc_{A}$, and $Fc_{P}$, the VSU prototype was tested according to the functioning scheme shown in Section 4.4 on the test video described in Section 5.2 and following the methodology therein. Figure 9 reports the relative results in terms of p(f) over time. Moreover, Table 7 summarises the tests results and compares them with the results of the same tests for the audio NN and picture NN models alone, while Figure 10 shows a comparison displaying the classification metrics.

Although the picture NN is the best in terms of recall, its usage without the audio NN causes too many FPs. Its adoption and exploitation according to the VSU prototype functioning scheme results in improvement of the system’s precision and accuracy, reducing FPs and avoiding FNs (the most dangerous outcome). Indeed, the test results prove that the picture NN is able to recognise all the alarms coming from the audio NN while blocking the propagation of FPs and FNs, thereby enchaining the precision of the whole system up to 100%. Moreover, the only misclassification took place in an ENT frame of the test video. Concerning the avoidance of FNs, this was ensured even by the picture NN on its own. However, in addition to the aforementioned motivations, using such a network would be too energy consuming, as demonstrated in Section 5.5.

**Figure 11:**
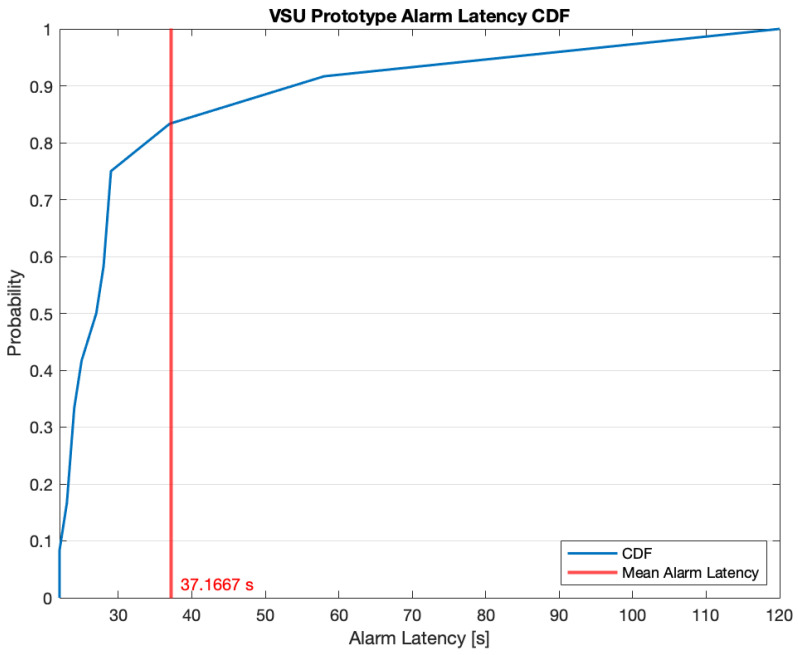
VSU prototype alarm latency CDF.

Figure 11 shows the Cumulative Distribution Function (CDF) related to the VSU prototype alarm latency owing to data acquisition, processing, and elaboration during the test video inferencing. The mean latency was 37.1667 s; from the latency CDF it can be noted that the latency is above the mean, with a probability of 0.16. However, the CDF additionally shows that the latency is less than 58 s with a probability of 0.92, while its maximum value was 120 s, meaning that the VSU prototype is able to signal fires in a timely fashion.

### 5.5. Current Drawn by the VSU Prototype

**Figure 12:**
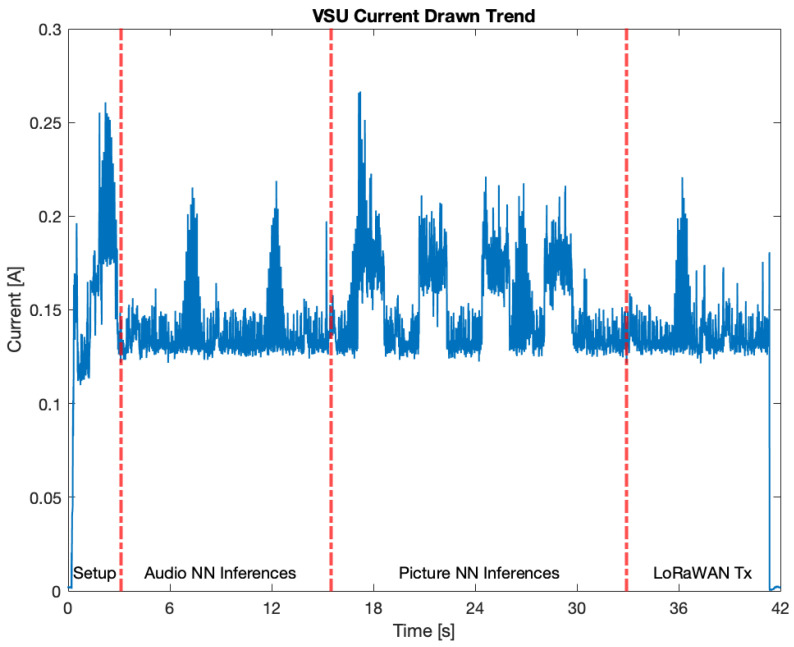
VSU prototype current drawn.

The last test for the VSU prototype was the measurement of its current drawn during operation. Because the VSU prototype is powered by exploiting a constant voltage input source, its power consumption is directly proportional to the current drawn. To this end, the prototype was tested on an ENT segment of the test video to assess a worst case scenario, and its current consumption was measured. The VSU prototype was powered via a dual channel bench power supply and the current draw was measured with a digital multimeter Agilent 34410A. The instrument was controlled via LabVIEW, and the readings were acquired at a sampling frequency of 5 Hz. Then, the sampled measurements were analysed by means of MATLAB, with the results provided in Figure 12. Four working periods can be identified: setup, audio NN inferences, picture NN inferences, and LoRaWAN transmission (i.e., “LoRaWAN Tx” in Figure 12). The setup phase lasted 3.12 s, during which the VSU executed the initial routines, requiring a mean current absorption of 144.3 mA. The audio NN inferences took a time interval of 12.38 s, during which the two inferences can be easily spotted, as current peaks (i.e., 215 mA and 218 mA) were experienced. During this period, a mean current absorption of 135 mA was required, and only the Cortex M4 core of the microcontroller was running. The picture NN inferences required 17.43 s. Similarly, the four inferences can be seen as current peaks (respectively, at 267 mA, 211 mA, 221 mA, and 216 mA) were present. Throughout this working phase, a mean current absorption of 150.8 mA was experienced, and only the Cortex M7 core of the microcontroller was running. Finally, the LoRaWAN transmission phase lasted 7.49 s, and the current peak of 221 mA related to the transmission can be seen. In this working period, a mean power absorption of 126.1 mA was needed, and the only active core was the Cortex M7. This test hints at the fact that, at least in this stage, the VSU prototype cannot only be battery-powered, and an energy harvesting system (e.g., photovoltaic panels) can be exploited in order to recharge a backup battery powering the system. Another alternative could be to provide the VSU prototype with duty cycling policies, though this would increase the alarm latency.

## 6. Conclusions

This paper proposed a prototype of an embedded IoT device implementing a VSU for early detection of forest fires. It uses two embedded ML algorithms, the former to deal with audio signals and the latter for pictures. To this end, two datasets were specifically gathered to train and test the models, and an ad hoc test video was created to test the system generalisation capability. Acoustic and visual data are acquired and sampled by the on-board microphones and camera, making the prototype fully stand-alone. Audio is continuously captured and analysed by the dedicated ML model; as soon as a fire is detected, pictures are taken that the ML model can assess the actual presence of fire. The working principle was proven to improve classification metrics, minimising FPs and especially FNs. More precisely, the VSU prototype achieved accuracy, recall, precision and F${}_{1}$ scores on the test video of 96.15%, 92.3%, 100%, and 96%, respectively, providing a mean alarm latency of 37.1667 s. In case of a fire, a LoRaWAN alarm is remotely sent to notify the personnel in charge. In future works, this prototype can be further developed and optimised from the point of view of both hardware components and software routines. Its classification features could be enhanced, and a reduction of its power consumption could be realised by adopting duty cycling policies. Moreover, the prototype could be equipped with data saving capabilities to collect an on-site dataset, which could help to further refine the ML models at the deployment site by adopting reinforcement learning paradigms.

## Figures and Tables

**Table 1:** Audio dataset description.

Class	Audio Type	Number of Samples	Total Time [s]
Fire	Clean Fire	272	1360
	Fire with Animals Sounds	272	1360
	Fire with Insects Sounds	272	1360
	Fire with Wind Noise	272	1360
	Additional Fire Samples	272	1360
	Recordings	72	360
No-Fire	Animals Sounds	276	1380
	Insects Sounds	200	1000
	Wind Noise	345	1725
	Rain Noise	211	1055
	Sundry Samples	400	2000

**Table 2:** Picture dataset description.

Class	Picture Type	Number of Samples
Fire	Daylight	1108
Fire	Night	1212
No-Fire	Snow	389
No-Fire	Daylight	1297
No-Fire	Fog	658
	Rain	
	Night	
No-Fire	Noise	396

**Table 3:** Hyperparameters and training parameters for audio NN #1 and audio NN #2.

		Audio	Audio
		NN #1	NN #2
Input Data:	Window Size [s]	4	2
Sliding Temporal	Sliding Step [s]	2	2
Windows	Sampling Frequency [kHz]	16	16
Feature Extraction: Mel-scaled Spectrograms	Temporal Frame Length [s]	0.050	0.050
	Sliding Step [s]	0.025	0.025
	FFT Points	1024	1024
	Filterbanks	40	40
	Filterbanks Lowest Frequency [Hz]	300	300
	Noise Floor [dB]	−52	−72
CNN Classifier	Architecture	1-D	2-D
	Convolutional-Pooling Layers	2	4
	Convolutional Neurons	8-16	8-16-32-64
	Fully Connected Layers/Neurons	1/64	1/64
	Dropout Layers/Rate	2/0.5	2/0.5
	Activation Function	ReLU	ReLU
Training Algorithm: Backpropagation	Loss Function	CCE	CCE
	Dataset Rate for Training Set	0.6	0.6
	Dataset Rate for Validation Set	0.2	0.2
	Dataset Rate for Test Set	0.2	0.2
	Learning Rate	0.005	0.005

**Table 4:** Hardware performance of the ML models.

Model	RAM	Flash	Execution	Overall
	Occupancy [kB]	Occupancy [kB]	Time [ms]	Accuracy [%]
Audio NN #1	16.8	72.5	6	90.890
Audio NN #2	38.0	119.6	89	95.375
Picture NN	283.0	162.6	43	87.495

**Table 5:** Test video results for audio NN model selection.

	Audio NN #1	Audio NN #2
${n_{A}}^{*}$	2	2
${th_{A}}^{*}$	0.808	0.940
TPs	12/13	12/13
TNs	11/13	11/13
FPs	2/13	2/13
FNs	1/13	1/13
Accuracy	88.5%	88.5%
Recall	92.3%	92.3%
Precision	85.7%	85.7%
F${}_{1}$ score	88.9%	88.9%

**Table 6:** Test video results for the picture NN model.

	Picture NN
${n_{P}}^{*}$	4
${th_{P}}^{*}$	0.823
TPs	13/13
TNs	9/13
FPs	4/13
FNs	0/13
Accuracy	84.6%
Recall	100.0%
Precision	76.5%
F${}_{1}$ score	86.7%

**Table 7:** Test video results for the VSU prototype compared with the results of the audio NN and picture NN models alone.

	Audio NN	Picture NN	VSU Prototype
TPs	12/13	13/13	12/13
TNs	11/13	9/13	13/13
FPs	2/13	4/13	1/13
FNs	1/13	0/13	0/13
Accuracy	88.50%	84.60%	96.15%
Recall	92.30%	100.00%	92.30%
Precision	85.70%	76.50%	100.00%
F${}_{1}$ score	88.90%	86.70%	96.00%

## Data Availability

The audio dataset, picture dataset, and test video can be found at [39,41,57], respectively.

## References

[1] San-Miguel-Ayanz J., Durrant T., Boca R., Maianti P., Liberta G., Artes Vivancos T., Jacome Felix Oom D., Branco A., de Rigo D., Ferrari D. (2021). Advance Report on Wildfires in Europe, Middle East and North Africa.

[2] Pham H.T., Nguyen M.A., Sun C.C. AIoT solution survey and comparison in machine learning on low-cost microcontroller. Proceedings of the 2019 International Symposium on Intelligent Signal Processing and Communication Systems (ISPACS).

[3] Merenda M., Porcaro C., Iero D. (2020). Edge machine learning for ai-enabled iot devices: A review. Sensors.

[4] Hu X., Li Y., Jia L., Qiu M. (2021). A novel two-stage unsupervised fault recognition framework combining feature extraction and fuzzy clustering for collaborative AIoT. IEEE Trans. Ind. Inform..

[5] Wang Y., Ho I.W.H., Chen Y., Wang Y., Lin Y. (2021). Real-time water quality monitoring and estimation in AIoT for freshwater biodiversity conservation. IEEE Internet Things J..

[6] Zualkernan I., Judas J., Mahbub T., Bhagwagar A., Chand P. An aiot system for bat species classification. Proceedings of the 2020 IEEE International Conference on Internet of Things and Intelligence System (IoTaIS).

[7] Bertocco M., Fort A., Landi E., Mugnaini M., Parri L., Peruzzi G., Pozzebon A. Roller Bearing Failures Classification with Low Computational Cost Embedded Machine Learning. Proceedings of the 2022 IEEE International Workshop on Metrology for Automotive (MetroAutomotive).

[8] Arif M., Alghamdi K., Sahel S., Alosaimi S., Alsahaft M., Alharthi M., Arif M. (2021). Role of machine learning algorithms in forest fire management: A literature review. J. Robot. Autom..

[9] Basu M.T., Karthik R., Mahitha J., Reddy V.L. (2018). IoT based forest fire detection system. Int. J. Eng. Technol..

[10] Deve K., Hancke G.P., Silva B.J. Design of a smart fire detection system. Proceedings of the IECON 2016-42nd Annual Conference of the IEEE Industrial Electronics Society.

[11] Alqourabah H., Muneer A., Fati S.M. (2021). A smart fire detection system using IoT technology with automatic water sprinkler. Int. J. Electr. Comput. Eng..

[12] Kadri B., Bouyeddou B., Moussaoui D. Early fire detection system using wireless sensor networks. Proceedings of the 2018 International Conference on Applied Smart Systems (ICASS).

[13] Baldo D., Mecocci A., Parrino S., Peruzzi G., Pozzebon A. (2021). A multi-layer lorawan infrastructure for smart waste management. Sensors.

[14] Sharma A., Singh P.K., Kumar Y. (2020). An integrated fire detection system using IoT and image processing technique for smart cities. Sustain. Cities Soc..

[15] Kinaneva D., Hristov G., Raychev J., Zahariev P. Early forest fire detection using drones and artificial intelligence. Proceedings of the 2019 42nd International Convention on Information and Communication Technology, Electronics and Microelectronics (MIPRO).

[16] Jiao Z., Zhang Y., Xin J., Mu L., Yi Y., Liu H., Liu D. A deep learning based forest fire detection approach using UAV and YOLOv3. Proceedings of the 2019 1st International Conference on Industrial Artificial Intelligence (IAI).

[17] Liu Z., Zhang K., Wang C., Huang S. (2020). Research on the identification method for the forest fire based on deep learning. Optik.

[18] Xu R., Lin H., Lu K., Cao L., Liu Y. (2021). A forest fire detection system based on ensemble learning. Forests.

[19] Benzekri W., El Moussati A., Moussaoui O., Berrajaa M. (2020). Early forest fire detection system using wireless sensor network and deep learning. Int. J. Adv. Comput. Sci. Appl..

[20] Harjoko A., Dharmawan A., Adhinata F.D., Kosala G., Jo K.H. (2022). Real-time forest fire detection framework based on artificial intelligence using color probability model and motion feature analysis. Fire.

[21] Zhang S., Gao D., Lin H., Sun Q. (2019). Wildfire detection using sound spectrum analysis based on the internet of things. Sensors.

[22] Li M., Zhang Y., Mu L., Xin J., Yu Z., Liu H., Xie G. Early Forest Fire Detection Based on Deep Learning. Proceedings of the 2021 3rd International Conference on Industrial Artificial Intelligence (IAI).

[23] Alves J., Soares C., Torres J.M., Sobral P., Moreira R.S. Automatic forest fire detection based on a machine learning and image analysis pipeline. Proceedings of the World Conference on Information Systems and Technologies.

[24] Chopde A., Magon A., Bhatkar S. Forest Fire Detection and Prediction from Image Processing Using RCNN. Proceedings of the 7th World Congress on Civil, Structural, and Environmental Engineering.

[25] Ya’acob N., Najib M.S.M., Tajudin N., Yusof A.L., Kassim M. (2021). Image processing based forest fire detection using infrared camera. J. Phys. Conf. Ser..

[26] Huang H.T., Downey A.R., Bakos J.D. (2022). Audio-Based Wildfire Detection on Embedded Systems. Electronics.

[27] Fort A., Peruzzi G., Pozzebon A. Quasi-Real Time Remote Video Surveillance Unit for LoRaWAN-based Image Transmission. Proceedings of the 2021 IEEE International Workshop on Metrology for Industry 4.0 & IoT (MetroInd4. 0&IoT).

[28] Oroceo P.P., Kim J.I., Caliwag E.M.F., Kim S.H., Lim W. (2022). Optimizing Face Recognition Inference with a Collaborative Edge–Cloud Network. Sensors.

[29] Dhou S., Alnabulsi A., Al-Ali A., Arshi M., Darwish F., Almaazmi S., Alameeri R. (2022). An IoT machine learning-based mobile sensors unit for visually impaired people. Sensors.

[30] Munteanu D., Moina D., Zamfir C.G., Petrea S.M., Cristea D.S., Munteanu N. (2022). Sea Mine Detection Framework Using YOLO, SSD and EfficientDet Deep Learning Models. Sensors.

[31] Peruzzi G., Galli A., Pozzebon A. A Novel Methodology to Remotely and Early Diagnose Sleep Bruxism by Leveraging on Audio Signals and Embedded Machine Learning. Proceedings of the 2022 IEEE International Symposium on Measurements & Networking (M&N).

[32] Hemdan E.E.D., El-Shafai W., Sayed A. (2022). CR19: A framework for preliminary detection of COVID-19 in cough audio signals using machine learning algorithms for automated medical diagnosis applications. J. Ambient. Intell. Humaniz. Comput..

[33] Pahar M., Niesler T. (2021). Machine Learning Based COVID-19 Detection from Smartphone Recordings: Cough, Breath and Speech. https://www.researchgate.net/publication/350673813_Machine_Learning_based_COVID-19_Detection_from_Smartphone_Recordings_Cough_Breath_and_Speech.

[34] da Silva B., Happi A.W., Braeken A., Touhafi A. (2019). Evaluation of classical machine learning techniques towards urban sound recognition on embedded systems. Appl. Sci..

[35] Ravi P., Syam U., Kapre N. Preventive detection of mosquito populations using embedded machine learning on low power iot platforms. Proceedings of the 7th Annual Symposium on Computing for Development.

[36] Antonini M., Vecchio M., Antonelli F., Ducange P., Perera C. (2018). Smart audio sensors in the internet of things edge for anomaly detection. IEEE Access.

[37] Fonseca E., Favory X., Pons J., Font F., Serra X. (2022). FSD50K: An open dataset of human-labeled sound events. IEEE/ACM Trans. Audio Speech Lang. Process..

[38] Piczak K.J. ESC: Dataset for Environmental Sound Classification. Proceedings of the 23rd Annual ACM Conference on Multimedia.

[39] Peruzzi G., Pozzebon A., Van Der Meer M. Audio Dataset. https://drive.google.com/file/d/15PQ-my8cA1blUIbAGRY8Jhq_d8Z7qim7/view.

[40] Dincer B. Wildfire Detection Image Dataset. https://www.kaggle.com/datasets/brsdincer/wildfire-detection-image-data.

[41] Peruzzi G., Pozzebon A., Van Der Meer M. Picture Dataset. https://drive.google.com/file/d/1QEAt4JiNxu5zZpXkWVnJm5sgtZm15Cf4/view?usp=share_link.

[42] Parri L., Parrino S., Peruzzi G., Pozzebon A. A LoRaWAN network infrastructure for the remote monitoring of offshore sea farms. Proceedings of the 2020 IEEE International Instrumentation and Measurement Technology Conference (I2MTC).

[43] Miranda I.D., Diacon A.H., Niesler T.R. A comparative study of features for acoustic cough detection using deep architectures. Proceedings of the 2019 41st Annual International Conference of the IEEE Engineering in Medicine and Biology Society (EMBC).

[44] Koike T., Qian K., Kong Q., Plumbley M.D., Schuller B.W., Yamamoto Y. Audio for audio is better? An investigation on transfer learning models for heart sound classification. Proceedings of the 2020 42nd Annual International Conference of the IEEE Engineering in Medicine & Biology Society (EMBC).

[45] Kutsumi Y., Kanegawa N., Zeida M., Matsubara H., Murayama N. (2023). Automated Bowel Sound and Motility Analysis with CNN Using a Smartphone. Sensors.

[46] Wang J., Liu K., Tzanetakis G., Pan J. (2020). Cooperative abnormal sound event detection in end-edge-cloud orchestrated systems. CCF Trans. Netw..

[47] Wu J., Liu Q., Zhang M., Pan Z., Li H., Tan K.C. (2021). HuRAI: A brain-inspired computational model for human-robot auditory interface. Neurocomputing.

[48] Howard A.G., Zhu M., Chen B., Kalenichenko D., Wang W., Weyand T., Andreetto M., Adam H. (2017). Mobilenets: Efficient convolutional neural networks for mobile vision applications. arXiv.

[49] Addabbo T., Fort A., Mugnaini M., Vignoli V., Intravaia M., Tani M., Bianchini M., Scarselli F., Corradini B.T. (2022). Smart Gravimetric System for Enhanced Security of Accesses to Public Places Embedding a MobileNet Neural Network Classifier. IEEE Trans. Instrum. Meas..

[50] Sineglazov V., Khotsyanovsky V. (2022). Camera Image Processing on ESP32 Microcontroller with Help of Convolutional Neural Network. Electron. Control Syst..

[51] Bilang J.M.D., Balbuena P.A.A.L., Villaverde J.F. Cactaceae Detection Using MobileNet Architecture. Proceedings of the 2021 IEEE 13th International Conference on Humanoid, Nanotechnology, Information Technology, Communication and Control, Environment, and Management (HNICEM).

[52] Audy Z. (2022). Analysis Quality of Corn Based on IoT, SSD Mobilenet Models and Histogram. J. Nas. Tek. Elektro Dan Teknol. Inf. Vol..

[53] Zheng W.C., Lee J.S., Sun Y.H. Development of AI-based Recycling Bins Using MobileNet-SSD Networks. Proceedings of the 2021 IEEE International Conference on Consumer Electronics-Taiwan (ICCE-TW).

[54] Rabano S.L., Cabatuan M.K., Sybingco E., Dadios E.P., Calilung E.J. Common garbage classification using mobilenet. Proceedings of the 2018 IEEE 10th International Conference on Humanoid, Nanotechnology, Information Technology, Communication and Control, Environment and Management (HNICEM).

[55] Kokilavani V. Intelligent Face Mask and Body Temperature Detection System using Machine Learning Algorithm. Proceedings of the 2022 International Conference on Innovative Computing, Intelligent Communication and Smart Electrical Systems (ICSES).

[56] Hossain D., Imtiaz M.H., Ghosh T., Bhaskar V., Sazonov E. Real-time food intake monitoring using wearable egocnetric camera. Proceedings of the 2020 42nd Annual International Conference of the IEEE Engineering in Medicine & Biology Society (EMBC).

[57] Peruzzi G., Pozzebon A., Van Der Meer M. Test Video. https://drive.google.com/file/d/1Hi2gs4mkrFibULaHfVDzgJZgVaVUYf6L/view?usp=share_link.

